# Innate immune response in acute critical illness: a narrative review

**DOI:** 10.1186/s13613-024-01355-6

**Published:** 2024-09-04

**Authors:** Laure Stiel, Alexandre Gaudet, Sara Thietart, Hélène Vallet, Paul Bastard, Guillaume Voiriot, Mehdi Oualha, Benjamine Sarton, Hatem Kallel, Nicolas Brechot, Louis Kreitmann, Sarah Benghanem, Jérémie Joffre, Youenn Jouan, la Commission de Recherche Translationnelle de la Société de Réanimation en Langue Française

**Affiliations:** 1 Department of Intensive Care Medicine, Groupe Hospitalier de la Région Mulhouse Sud Alsace, Mulhouse, France; 2 Lipness Team, INSERM Research Team, LNC UMR 1231 and LabEx LipSTIC, University of Burgundy, Dijon, France; 3 CHU Lille, Department of Intensive Care Medicine, Critical Care Center, Univ. Lille, 59000 Lille, France; 4 CIIL (Centre d’Infection et d’Immunité de Lille), Institut Pasteur de Lille, U1019-UMR9017, 59000 Lille, France; 5 Département de Gériatrie, Sorbonne Université, Assistance Publique-Hôpitaux de Paris (AP-HP), Hôpital Pitié-Salpêtrière, Paris, France; 6 Inserm, PARCC U970, F75, Université Paris Cité, Paris, France; 7 Department of Geriatric Medicine, Sorbonne Université, Assistance Publique-Hôpitaux de Paris (APHP), Hôpital Saint Antoine, Paris, France; 8 INSERM UMR1135, Centre d’immunologie et des Maladies Infectieuses, Sorbonne Université, Paris, France; 9 Laboratory of Human Genetics of Infectious Diseases, Necker Branch, INSERM U1163, Necker Hospital for Sick Children, Paris, France; 10 Imagine Institute, University of Paris, Paris, France; 11 Pediatric Hematology-Immunology and Rheumatology Unit, Necker Hospital for Sick Children, Assistance Publique-Hôpitaux de Paris (AP-HP), Paris, France; 12 Service de Médecine Intensive Réanimation, Hôpital Tenon, Hôpitaux de Paris, Paris, France; 13 Centre de Recherche, Saint-Antoine UMRS_938, INSERM, Sorbonne Université, Assistance Publique, Paris, France; 14 Pediatric Intensive Care Unit, Necker Hospital, APHP, Centre-Paris University, Paris, France; 15 Service de Réanimation Polyvalente Purpan, Centre Hospitalier Universitaire de Toulouse, Toulouse, France; 16 ToNIC Lab (Toulouse NeuroImaging Center) INSERM/UPS UMR 1214, 31300 Toulouse, France; 17 Service de Réanimation, Centre Hospitalier de Cayenne, Guyane, France; 18 Service de Médecine Intensive Réanimation, Sorbonne Université, Hôpitaux Universitaires Pitié Salpêtrière- Charles Foix, Assistance Publique-Hôpitaux de Paris (AP-HP), Paris, France; 19 Center for Interdisciplinary Research in Biology (CIRB)-UMRS, INSERM U1050-CNRS 7241, College de France, Paris, France; 20 Centre for Antimicrobial Optimisation, Department of Infectious Disease, Faculty of Medicine, Imperial College London, London, W12 0HS UK; 21 ICU West, The Hammersmith Hospital, Du Cane Road, London, W12 0HS UK; 22 Service de Médecine Intensive Réanimation, Hôpital Cochin, Assistance Publique-Hôpitaux de Paris (AP-HP), Paris, France; 23 Service de Réanimation Médicale, Hôpital de Saint Antoine, Assistance Publique-Hôpitaux de Paris (AP-HP), Paris, France; 24 Centre de Recherche Saint Antoine INSERM, U938, Sorbonne University, Paris, France; 25 Service de Médecine Intensive Réanimation, CHRU Tours, Tours, France; 26 Services de Réanimation Chirurgicale Cardiovasculaire et de Chirurgie Cardiaque, CHRU Tours, Tours, France; 27 INSERM, U1100 Centre d’Etudes des Pathologies Respiratoires, Faculté de Médecine de Tours, Tours, France

**Keywords:** Innate immunity, Immunothrombosis, Immunosenescence, Trained immunity, Acute critical illness

## Abstract

**Background:**

Activation of innate immunity is a first line of host defense during acute critical illness (ACI) that aims to contain injury and avoid tissue damages. Aberrant activation of innate immunity may also participate in the occurrence of organ failures during critical illness. This review aims to provide a narrative overview of recent advances in the field of innate immunity in critical illness, and to consider future potential therapeutic strategies.

**Main text:**

Understanding the underlying biological concepts supporting therapeutic strategies modulating immune response is essential in decision-making. We will develop the multiple facets of innate immune response, especially its cellular aspects, and its interaction with other defense mechanisms. We will first describe the pathophysiological mechanisms of initiation of innate immune response and its implication during ACI. We will then develop the amplification of innate immunity mediated by multiple effectors. Our review will mainly focus on myeloid and lymphoid cellular effectors, the major actors involved in innate immune-mediated organ failure. We will third discuss the interaction and integration of innate immune response in a global view of host defense, thus considering interaction with non-immune cells through immunothrombosis, immunometabolism and long-term reprogramming via trained immunity. The last part of this review will focus on the specificities of the immune response in children and the older population.

**Conclusions:**

Recent understanding of the innate immune response integrates immunity in a highly dynamic global vision of host response. A better knowledge of the implicated mechanisms and their tissue-compartmentalization allows to characterize the individual immune profile, and one day eventually, to develop individualized bench-to-bedside immunomodulation approaches as an adjuvant resuscitation strategy.

## Introduction

During acute critical illness (ACI), from sepsis to trauma, innate immunity is triggered instantaneously. Activation of innate immunity not only aims at eliminating pathogens, but also to avoid tissue damage and alleviate potentially harmful unregulated inflammation. While innate response represents a keystone of patients’ survival, aberrant activation may participate in the occurrence of organ failures.

This narrative review aims to provide a non-specialist audience with recent and relevant discoveries in the ACI-associated innate immune response field. Immunology is a rapidly growing scientific field, and advances in knowledge and technologies have fueled significant therapeutic progress in numerous medical areas, but not yet in intensive care. Nevertheless, intensivists now use diverse therapies to modulate the inflammatory response as part of clinical trials, and a large proportion of patients admitted in ICU undergo an immunomodulators or immunosuppressive treatments. This way, intensivists need to understand the underlying basic concepts supporting new personalized strategies.

Therefore, we will describe the ubiquitous and organ-specific pathophysiological mechanisms of activation of the innate immune response during ACI, with a focus on myeloid and lymphoid cellular effectors. Moreover, interaction and integration of innate immunity with other host defense systems will also be considered through immunothrombosis immunometabolism and trained immunity. The specificities of the immune response in children and elderly will also be discussed. In each section, we will first describe the known or putative molecular and cellular mechanisms involved in the activation of innate immunity.

### Initiation of innate immunity

Initiation of the innate immune response requires the recognition of the aggression through evolutionarily conserved signals. During ACI, the accurate identification of these signals is essential to recognize the danger and stop its propagation.

#### PAMPs and DAMPs

Inflammation frequently results from the exposure of immune cells to pathogen-associated molecular patterns (PAMPs), which are conserved motifs expressed by microorganisms. Damage-associated molecular patterns (DAMPs), mainly represented by residues of necrotic cells produced in inflammatory processes, including non-infectious aggressions, as observed in trauma or burns [1], can alternatively trigger the immune response.

PAMPs are structurally diverse and exhibit a relative specificity to various pathogen groups (*e.g.* lipopolysaccharide (LPS) for gram-negative bacteria or mannane antigen for fungi). Thus, identifying conserved pathogenic motifs by innate immune cells enables the establishment of an initial level of specificity in response to the invading pathogen. Consequently, differential activation of the immune cells are observed depending on the nature of the recognized pathogen, *e.g*., cellular recognition of viral patterns results in local induction of type I interferon synthesis and, secondarily, activation, proliferation, and differentiation of cytotoxic T lymphocyte clones in the secondary lymphoid organs.

DAMPs are thus initiators of the so called ‘sterile inflammation’, and mechanistically lead to initiation of inflammatory cascades, in a similar -but not systematically redundant- ways to PAMPs [1, 2]. DAMPs encompass a wide variety of molecules, from both intracellular compartments to extracellular components. Prototypical DAMPs from intracellular origin are the DNA binding protein HMGB1, DNA, ATP, RNA, and mitochondrial components from stressed/damaged cells are known DAMPs able to promote inflammatory response once release out of their physiologic compartment. The term ‘alarmin’ is also sometimes employed to refer to these endogenous molecules from intracellular origin, and also include specific molecules like HMGB1, IL-33, S100, heat shock proteins that have similar roles [3]. Extracellular matrix components, such as hyaluronic acid or heparan sulfate are also classical DAMPs, released by matrix degradation during tissue injury. DAMPs are of high interest in ACI as they are associated with commonly seen pathophysiological mechanisms: ischemia–reperfusion mechanisms, tissue injury (trauma). Moreover, link with DAMPs released in critically ill patients have been associated with poor outcomes [4].

#### PRMs—pentraxins

Humoral part of innate immunity includes soluble recognition molecules (PRMs) that recognize PAMPs and DAMPs.

PRMs notably encompass the pentraxin family, consisting of proteins that possess a shared domain and are constructed from monomers organized into multimeric structures with a disc-like form. A defining feature of this family is a conserved sequence of 205 amino acids located at the C-terminal, referred to as the pentraxin domain. Based on the overall length of their protein sequences, the pentraxin family can be divided into two subgroups: short and long pentraxins. Short pentraxins include proteins like C-reactive protein (CRP, also known as pentraxin 1 or PTX1) and serum amyloid P component (SAP, also known as pentraxin 2 or PTX2), while long pentraxins encompass proteins such as neuronal pentraxin 1 (NPTX1), neuronal pentraxin 2 (NPTX2), neuronal pentraxin receptor (NPTXR), pentraxin 3 (PTX3), and pentraxin 4 (PTX4) [5, 6]. Numerous studies have shed light on the specific roles played by certain members of the pentraxin family. Notably, CRP and SAP have been recognized for their regulatory functions within the human immune system including defending against pathogens, linked to their capability to attach to a variety of bacteria, fungi, and viruses, thereby bolstering the innate immune responses against these pathogens [5, 7–10]. Moreover, pentraxins bind to phospholipids and certain nuclear ribonucleoproteins within apoptotic cells, facilitating their non-inflammatory clearance. C-reactive protein, SAP, and PTX3 engage with diverse complement molecules, thereby amplifying their recognition capabilities, and facilitate the phagocytosis of microbes and apoptotic cells through interactions with FcγR [11, 12]. The two-sides of the discoid structure of pentraxins seem to exhibit complementary features of the aforementioned functions. This is notably illustrated in CRP, with one side being involved in the activation of the classical complement pathway, thereby facilitating phagocytosis, whiles the other side interacts with bacterial cell walls phosphorylcholine, thereby leading to pathogen clearance [13, 14].

#### PRRs

PAMPs and DAMPs are recognized by tissue-resident immune cells (mast cells, macrophages and tissue dendritic cells -tDCs-) but also by epithelial and endothelial cells, expressing pattern recognition receptors (PRRs). Once activated, these cells release inflammatory mediators (pro-inflammatory cytokines, lipid derivatives…), activating endothelial cells and initiating the vascular phase of the inflammatory response.

PRRs can interact with numerous antigenic patterns, including LPS, peptidoglycan, viral RNA or bacterial DNA. Their localization (on cellular or endosomal membranes, in the cytosol or secreted [15]) generally fits with the cellular compartment in which the recognized pathogens are found.

While PRRs play a key role in initiating and amplifying the immune response, which is essential for the early recognition and control of aggression, they can also turn into a dysregulated response, subsequently leading to organ failure through cellular and tissue damage.

Membrane PRRs, known as Toll-like receptors (TLRs) induce cell activation via NF-kB-dependent signaling cascades (modulation of genes coding for pro-inflammatory molecules or co-stimulatory molecules essential for activating the adaptive response). These receptors have various ligands, including peptides, carbohydrates, lipids, DNA, and RNA. Several types of TLRs are observed in primary effectors of the immune response like neutrophils, including TLR1, − 2, − 3, − 4, − 5, − 6, − 8, and − 10, each of them recognizing various ligands. The engagement of these TLRs contributes to the initiation of the innate immune response through various mechanisms such as the TRIF pathways, involved in the production of type I interferon and pro-inflammatory mediators following the activation of TLR3 and 4 [16]. This priming of innate response secondarily stimulates the secretion of cytokines by innate immune cells and enhances phagocytosis [17–19].

C-type lectin receptors (CLRs) like Dectin-1 are major PRRs meaning they induce the phagocytosis of recognized pathogens. Dectin-1 is notably involved in the internalization and killing of fungi, through fungal β-glucans antigens recognition [20, 21].

Nucleotide-binding Oligomerization Domain (NOD) 1 and NOD2 are other PRRs localized in the cytosol, interacting with peptidoglycan-related molecules. They belong to the larger family of NOD-like receptors (NLRs). The stimulation of NOD2 by its ligand triggers the secretion of IL-8 in neutrophils. Activation of cytosolic NLRs also leads to cell activation (synthesis of the pro-inflammatory cytokine IL-1) [22, 23].

#### Signal integration & amplification during innate & inflammatory response

This wide range of PRRs allows specific identification of the nature of the threat, and, at the cellular level, multiple signals from PAMPs and DAMPs are integrated and regulated to produce tightly modulated effector response [24–26]. These varied pathways contribute to the diversity of observed profiles based on the origin of the inflammatory response. Recent research highlights that patient phenotypes predominantly rely on the source of the initial aggression [27]. Interestingly, beyond recognition by PRR of foreign versus endogenous ligands, many immune & barrier cells can also sense a harmful / stressful environment both at extra and intracellular levels. This is notably done by the cytosolic multiproteic complexes called ‘inflammasomes’ and especially the NLRP3 inflammasome [28]. Once assembled and activated, NLRP3 inflammasome lead to caspase 1 activation that can produce mature IL-1β, but also leading to necroptosis, an inflammatory death cell [29]. Thus, important cellular perturbation resulting from an initial insult can lead to inflammatory death, locally perpetuating inflammatory signal. Given that other inflammatory cell deaths have been discovered during the last decades, it has been proposed that inflammation and inflammatory cell death could self-perpetuate in organs after initial insult, thus contributing to organ failure [30].

### Effectors of innate immunity

**Fig. 1:**
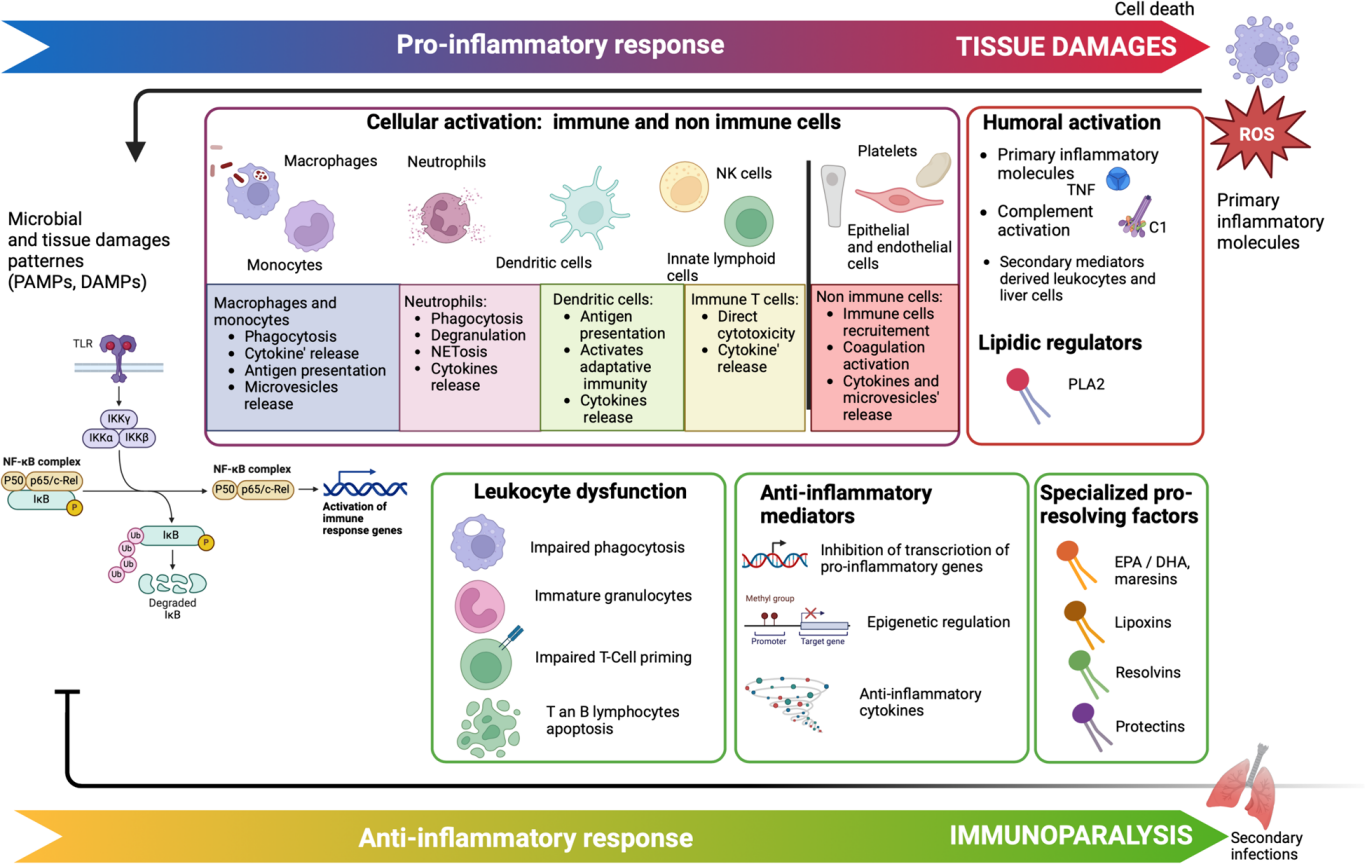
Innate immune response. Innate immune response is characterized by both proinflammatory (in red, top of the figure) and anti-inflammatory (in green, bottom of the figure) responses. Pro-inflammtory response can results in cellular damages forming DAMPs, that themselves trigger inflammation pathways. Anti-inflammatory response can induce immunoparalysis, resulting in secondary infections. The balance of this host response, its duration and its intensity are dependent of multiple factors: type of aggression on one hand, host factors (genetic, immune state, medical history, age …) on the other hand. *C1* complement 1, *DAMPs* danger associated molecular patterns, *DHA* docosahexaenoic acid, *EPA* eicosapentaenoic acid, *IL* interleukin, *NETosis* release of NETs, Neutrophil Extracellular Traps, *PAMPs* pathogen associated molecular patterns, *PLA2* phospholipase 2, *ROS* reactive oxygen species, *TLR* toll like receptor, *TNF* tumor necrosis factor

Recognition of danger signals induces rapid mobilization of immune cells from the bone marrow to the injury site. Immune activation aims simultaneously to limit tissue invasion and initiates the resolution of inflammation (Fig. 1).

This section focuses on the understanding of myeloid and lymphoid activation and dysfunction, and their potential consequences for clinicians through innovative therapy targeting innate immune cells. A recently published review thoroughly describes the humoral part of innate immunity, and will thus not be reviewed here [31].

#### Myeloid cells

##### From host defense to tissue damage

Myeloid cells, including monocytes, macrophages, neutrophils, and DCs, are the first cells activated in early response to a danger signal. Neutrophils are sensitive to different inflammatory, infectious or physical signals which induce their release from bone marrow, intravascular and transendothelial migration to the aggression site [32, 33]. Phagocytosis was long considered as the primary function of macrophages and neutrophils on the injury site, especially considering infections [34]. Phagocytosis occurs through different mechanisms. One is oxygen-dependent and mediated by the reactive oxygen species (ROS). ROS are highly toxic, and capable of bacterial destruction but also participate to tissue injuries [35]. High levels of ROS are associated with early death and post-aggressive immunodepression, and secondary infections. Mitochondria are the main source of ROS production, and therefore appear as key components of the immune regulation occurring in critically patients. It seems noteworthy to mention that mitochondrial ROS not only participate to direct bacterial killing, but upregulate the synthesis of pro-inflammatory mediators as well, through activation of the NLRP3 inflammasome [36]. Unbalanced ROS production in myeloid cells may directly inflict local damages to mitochondrial proteins and DNA, thereby leading to significant impairment of their function during the immune response [37].

This example illustrates the complex functional ambiguity of myeloid cells during ACI: while their role in host defense is essential for survival, their activation can also become aberrant and participate in organ dysfunctions [38]. Thus, overstimulation of the bone marrow also triggers the release of immature monocytes and neutrophils, called myeloid-derived suppressive cells (MDSCs). Circulating immature granulocytes present altered effector capacity *(i.e.* pathogen recognition, reduced phagocytosis capacities,) and their abundance is associated with early mortality and secondary immunosuppression [39–41]. MDSCs also participate to tissue hypoperfusion through microvessels obstruction due to their immature rheological properties [42]. Monocytic MDSCs are characterized by a decreased surface-expression of human leucocyte antigen HLA-DR, commonly associated with nosocomial infections and secondary-immunosuppressive state [43].

##### Lipid mediators of innate immunity

In response to primary inflammatory mediators (IL-1, Tumor Necrosis Factor -TNF-,) myeloid cells synthesize phospholipase A2 (PLA2) that transforms membrane phospholipids in arachidonic acid. Released free arachidonic acid can be further metabolized (i) by cyclooxygenase (COX) 1 and 2 to prostaglandins (PG) and thromboxanes (TXA), and (ii) by lipoxygenase (LO) to leukotrienes (LK) and lipoxins [44]. These molecules are named eicosanoids. They are rapidly metabolized, resulting primarily in a local action due to their short lifespan [45].

Eicosanoids are critical actors in the regulation of inflammation [28] during ACI, by regulating vasodilatation-vasoconstriction balance, vascular leakage, and platelet activation. One example in ACI is the elevation of circulating Platelet-activating factor (PAF), during critical phase of dengue hemorrhagic fever that potentially promotes major capillary leak syndromes [46]. Eicosanoids and other lipids are also implicated in the resolution of injury through anti-inflammatory effects [47]. Lipoxins can inhibit macrophages and neutrophils recruitment. These specialized pro-resolving mediators (SPMs) include other lipid molecules like omega-3 derived fatty acids that have been isolated in inflammatory exudates [48]. SPMs also participate to bacterial clearance and efferocytosis. Because of their immunomodulation properties, nutritional supplementation of omega-3 fatty acids was proposed in septic patients, but did not improve mortality [49].

Inhibition of the formation of eicosanoids by aspirin or non-steroidal anti-inflammatory drugs is a classical therapy to prevail fever or pain. Nevertheless, eicosanoids synthesis is very dynamic and compartmentalized, with tight organ-specific regulation. This complexity may explain the failure of clinical trial testing inhibition of COX2 by ibuprofen during septic shock [50]. A more extensive understanding of the activated pathways and the identification of the side products of biosynthesis using new tools like mass-spectrometry lipidomic profiling is needed [51, 52].

##### Interplay between macrophages and neutrophils

Recent advances in technologies exploring immunity helps uncovering novel functions of myeloid cells. Various subpopulations of macrophages have been identified across different tissues and exhibit various functions beyond phagocytosis [38]**.** Ischemia–reperfusion**,** which is observed in a wide range of ACI, is characterized by an initial restriction of oxygen supply to an organ before perfusion is restored. Hypoxia induces endothelial dysfunction [53] and results in the activation of various cellular cell death programs including NETosis (release of Neutrophil Extracellular Traps), apoptosis and autophagy [54]. Like cytokine storms, excessive release of NETs and apoptotic cells may exacerbate the inflammatory state during sterile aggressions, thus participating in the development of acute respiratory syndrome (ARDS) as observed in trauma patients [55]. Surprisingly, experimental studies have shown that increased neutrophil lifespan is associated with a deleterious impact [56]. This could be explained by the reduced clearance of NETs and apoptotic cells by macrophages, called efferocytosis [57]. Cytoskeletal modifications of macrophages are required for this function. Experimental studies have identified the inhibition of AMP-activated protein kinase (AMPK) as a significant efferocytosis contributor [58]. Inhibition of AMPK activity is observed during ARDS [59]. Restoration of AMPK activity, and thus macrophage function, represents a promising target for reducing lung inflammation [57].

##### Interaction with adaptive immunity

Overall, the immune response is highly dynamic and myeloid cells interact with other cells. Neutrophils can directly activate DCs through DC-SIGN receptors expressed on the surface of immature DCs and Mac-1, and this interaction is essential for both cellular functions [60]. DCs are responsible for the initiation of antigen-specific immune responses. The direct interaction with neutrophils orientates the polarization of lymphocytes to a Th1 phenotype [61], but also requires a favorable microenvironment including the presence of TNFα. DCs could thus play a major role in immune regulation, and represent a potential effector for the development of therapeutic vaccines [62]. Myeloid cells are also able to stimulate or inhibit B-lymphocytes in the lymphoid organs, depending on their microenvironment.

#### Innate lymphoid cells and innate T cells

Immune cells of lymphoid origin differ from myeloid cells during their ontogeny in the bone marrow. The common lymphoid progenitor (CLP) first emerges from hematopoietic stem cells (HSCs). Then, this CLP can remain in the bone marrow and engage in the innate lymphoid lineage, or engage in T cell fate, by reaching the thymus and pursuing their dedicated specific T cell maturation.

##### Innate lymphoid cells

ILCs are a peculiar and heterogenous population of immune cells. However, they do not express a diversified antigen receptor, therefor excluding them from T and B lymphocytes families, and can rapidly provide their effector function. Thus, they are considered to be part of the innate immune system [63]. From a functional point of view, ILCs are often considered as the innate counterpart of conventional T cells and have emerged as key players in the early orchestration of the immune response, particularly at the barrier sites (lung, skin, gut) [64]. Different subsets have been described, schematically mirroring those known for T cells: some subsets are known to have “helper” properties, -in a similar way to CD4^+^ T helpers-, by producing cytokines according to the type of aggression (termed ILC1, 2 and 3). Similarly, the so-called “Natural-Killers” cells are classified as ILC and mirror the cytotoxic CD8^+^ T cells.

ILCs are usually not the first responders among the innate immune arm but are instructed by signals (cytokines) provided by the cells that contact pathogens/aggressors. However, their strategic position at barrier sites allows them to be rapidly informed of local modification in the environment and potential threats. Indeed, ILCs are mostly tissue-resident cells with the notable exception of circulating NK cells that can represent up to 15% of circulating lymphocytes. Tenrichment in different subsets of ILCs varies according to the tissue considered [64]. Besides aggression, ILCs also contribute locally to the maintenance of homeostasis, especially in the gut, through the control of epithelial integrity and interaction with the microbiome [64, 65]. Last, ILCs also shape the adaptive response, either through soluble mediators acting on adaptive cells or by direct cellular interaction with adaptive cells [66].

In vivo studies in mice using viral, bacterial and fungal models of lung, skin, and gut infections have shown the involvement of ILCs in the control and resolution of infection [64, 67, 68]. Protection is thus conferred by secondary signals released by ILCs to induce appropriate immune response: IL-17 and IL-22 produced in response to extra-cellular pathogens induce local production of anti-microbial peptides and recruitment of neutrophils; IFNγ produced during infection by intra-cellular pathogens induces the recruitment of phagocytes etc. NK cells also have cytotoxic properties, that confer protection notably against intra-cellular infections (but also against tumoral cells). Regarding pathogens experimentally evaluated in this field, many are potentially relevant in the setting of ACI: *influenza, S. pneumoniae, K. pneumoniae, C. difficile, C. albicans*. It is also noteworthy that evidence exists for the involvement of ILCs in tissue repair after acute injury, notably ILC3 and ILC2, for example after *influenza* pneumonia [65].

However, to date in human, data from patients during disease are scarce, especially for ILC1, 2 and 3 subpopulations, recently discovered and technically more complex to analyze. Studies, due to obvious ethical and technical limitations, are mostly descriptive and limited to chronic inflammatory condition (i.e. asthma, psoriasis, [69, 70]), apart from the recent publication of ILCs alteration in function and frequency in peripheral blood during COVID-19 [71]. Data accumulated regarding NK cells in human, however, are more extensive, revealing alteration in blood frequency and function of NK cells during sepsis, but associations with outcome are controversial [72]. It is reasonable to hypothesize that NK cells implication in sepsis might vary according to the considered stage of the disease (i.e., early course of sepsis versus post-aggressive immunesuppressive phase).

At the end, the discovery of these cells and data accumulating regarding their implication in numerous physiological and pathological settings are redefining our way of considering the innate immune response and could be a game-changer in a near future.

##### Innate T cells

The term “innate T cells” (ITCs), is used to describe subpopulations of T cells that are endowed with specific properties and functions, differing from conventional adaptive T cells, and ontogenetically, they differentiate from conventional arm during thymic maturation [73]

ITCs have unique and complementary properties compared to ILCs and conventional adaptive T cells, and they are thus strategically poised at the interface between adaptive and innate immunity, which they can both modulate and shape according to the type of threats they face. Moreover, ITCs are enriched at barrier sites where they can exert their versatile functions ranging from initiation and amplification of the immune response to tissue repair [74]. All these properties make ITCs potentially attractive targets in the immunopathology of ACI. The recent development of specific tools for their detection led to a growing body of evidence of their implication in ACI, notably Mucosal-Associated Invariant T (MAIT) cells, as exemplified by the COVID-19 pandemic [75–77].

Innate T cells are also described as “preset T cells”, capable of rapid activation, similar to innate immune cells [78]. Moreover, unlike conventional adaptive T cells, they do not need to go through a clonal expansion phase after being stimulated. Two main lineages of ITCs are described: invariant Natural Killer T (iNKT) cells and MAIT cells. A third population can be added to the “innate T cells” subgroup, comprising some T cells harboring a TCR built with specific γ and δ chains (mainly, in humans those harboring Vγ9 Vδ2 chains) [79].

Through their TCR, MAIT and iNKT cells recognize small non-peptidic antigens presented by non-polymorphic MHC-related molecules: thus, ITCs harbor TCR and recognize antigens invisible to conventional T cells -that recognize small peptides. Moreover, ITCs can be activated through TCR-independent stimulation, notably via cytokines.

Upon stimulation, Innate T cells can exert multiple immune functions, from cytotoxicity to tissue repair [74]. Akin to ILC and conventional T cells, some effector functions of ITCs can also be considered as “helpers”, depending on the cytokines they produce.

A substantial body of evidence has demonstrated the implication of ITCs in various experimental models of ACI. Relevant models of bacterial and viral pneumonias have demonstrated the beneficial role of ITCs during infection through the orchestration of immune response and tissue repair [80, 81]. However, how these cells could be implicated in dysregulated host response as observed in ARDS and other ACI remains largely unknown and justify in-depth analysis of these cells.

In clinical settings, the implication of ITCs has been observed in patients with sepsis, severe *influenza*, and severe COVID-19 patients [75–77, 82, 83]. Both iNKT and MAIT cells frequencies in the blood were drastically reduced in these conditions, while presenting activation markers. Association with patients’ outcomes has also been explored, highlighting a correlation between MAIT cell persistent deficiency and the frequency of nosocomial infections [82]. Moreover, in severe COVID-19, MAIT cell activation was associated with poor outcome in three studies while being associated with better outcome in another study [75–77]. These discrepancies, possibly due to differences in patients’ severity suggest that MAIT cells might have dual and opposite functions, according to environmental cues [84].

ITCs are already considered as attractive targets for immune intervention, notably because of their vast array of functions and their specificity for public antigens presented by conserved and non-polymorphic molecules. Thus, several clinical trials are already ongoing in other medical fields (especially oncology) [85].

### Immunothrombosis

In response to aggression, vascular cells, including innate immune cells, platelets, and endothelial cells are activated and trigger innate immunity and coagulation pathways. The crosstalk between immunity and coagulation was thus recently defined by the term “immunothrombosis” [86]. It refers to an innate intravascular immune mechanism that recognizes and contains the injury through the activation of innate immunity and coagulation, leading to the formation of “protective” thrombi in microvessels [86]. Unbalanced and dysregulated immunothrombosis results in inadequate host response during infectious diseases and sterile ACI like myocardial infarction, stroke or trauma.

**Fig. 2:**
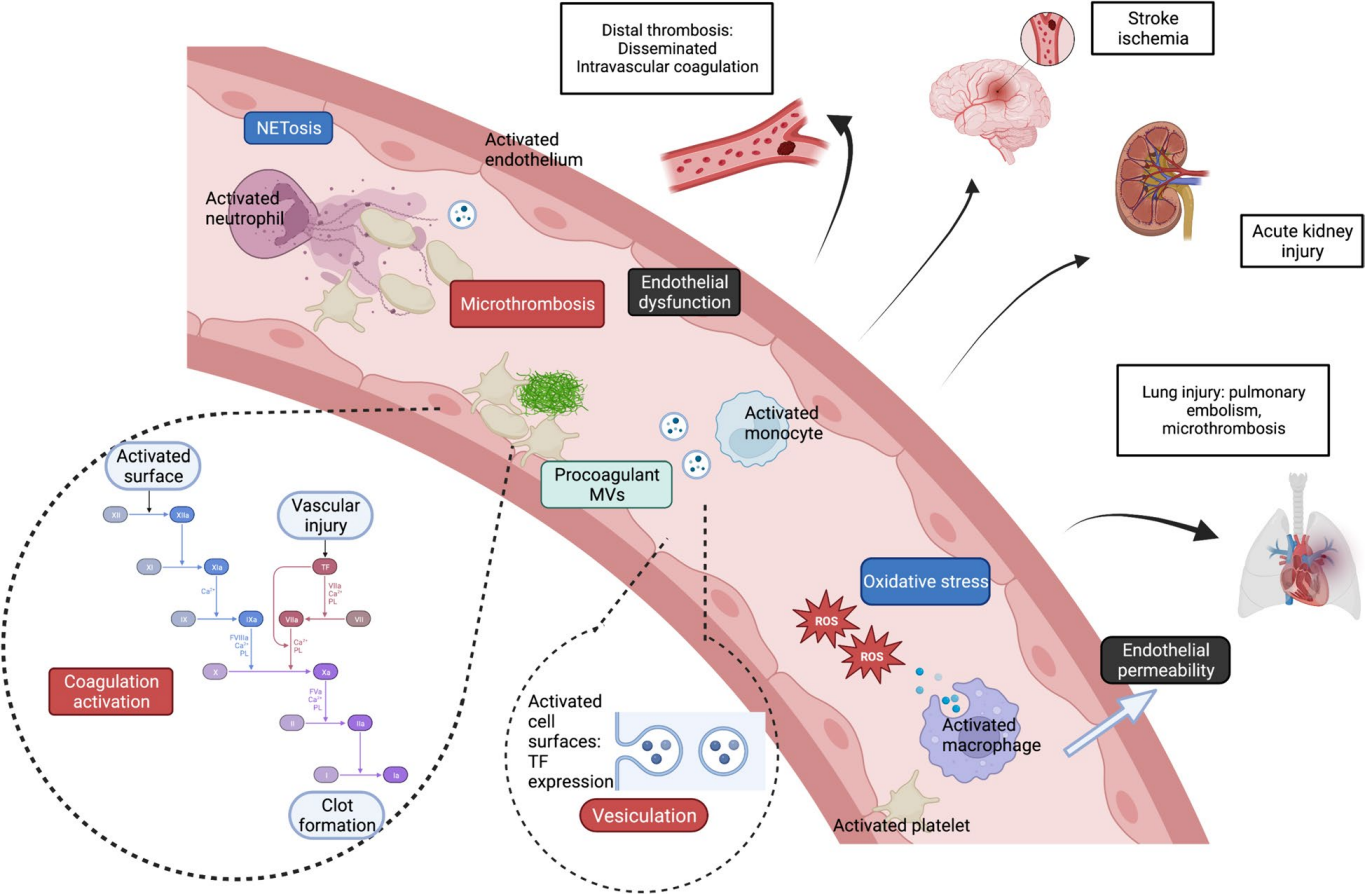
Immunothrombosis in acute critical illness. Immunothrombosis is a local defense mechanism that can be dysregulated during ACI, inducing an hyper-inflammatory and hyper-oxidative state. Impaired immunothrombosis is notably mediated by neutrophils and endothelial dysfunction, activated leukocytes and platelets. Dysregulated immunothrombosis results in microthrombi formation, responsible of various organ dysfunction during ACI. *MVs* microvesicles, *NETs* neutrophil extracellular traps, *ROS* reactive oxygen species, *TF* tissue factor

Neutrophils recruited to the site of infection in response to chemoattractant molecules like CXCL2 release the content of their granules and ROS. Platelets are activated through the binding of glycoprotein (Gp) Ib-V-IX to Willebrand Factor [86]. They release the content of their granules, including RANTES, PF4 and CD40L [87] and interact with myeloid cells via direct binding to different receptors like Mac-1 (Macrophage 1 antigen) expressed on myeloid cells [88]. Monocytes, platelets and neutrophils also release microvesicles (MVs) [89, 90]. Tissue factor (TF) expression on the surface of the activated cells, MVs, NETs, and some PAMPS and DAMPS contribute to the coagulation pathway activation to contain the zone of injury. The interactions between coagulation and immunity thus represent a first line of defense. During ACI, the severity of the injury and the inadequate host response with deregulated immunothrombosis promote a hyper-inflammatory and hyper-oxidative state [91]. Subsequently, the previously described impaired myeloid functions, including neutrophil CCR2 expression, lead to their accumulation in organs, thus contributing to multiple organ failure [92]. The systemic inflammation also induces an exaggerated and uncontrolled NETosis, participating in capillary occlusions. Endothelial dysfunction promotes leukocyte and platelet adhesion, coagulation activation, and fibrinolysis inhibition. Ultimately, uncontrolled thrombin formation (excessive activated thrombin FIIa formation) leads to disseminated microthrombi, capillary occlusions, and subsequent impaired organ perfusion. The main clinical issue revealing the deregulated immunothrombosis is disseminated intravascular coagulation observed during different ACI as diverse as trauma, septic shock or obstetrical diseases. Exaggerated immunothrombosis can also be a compartmentalized phenomenon, for example in Sars-CoV2-infected lungs [93–95] and also in non-COVID ARDS [96] or after surgery [97] (Fig. 2).

Numerous randomized clinical trials have failed to demonstrate the benefits of anticoagulation and anti-inflammatory therapies during ACI, especially in septic shock. The better comprehension of the crosstalk between innate immune cells and non-immune effectors offers new hopes for developing targeted therapeutics, *i.e. by targetting* NETs. The pathogenicity of NETs is no well demonstrated in animal models. Besides, NETs are implicated in tissue damages in a relevant murine model of lesional pulmonary oedema [55]. The administration of anti-histone antibodies prevented the extension of pulmonary lesions. In another model of acute lung injury in mice, DNase and inhibitor of neutrophil elastase also attenuated lung injury [98].

### Trained immunity

Trained immunity defines the innate immunological memory orchestrated by epigenetic reprogramming. This concept encompasses changes in gene expression and cellular physiology without permanent genetic changes, such as epigenetic modifications. These chemical changes, including DNA methylation, histone modification, and RNA-associated silencing can affect gene expression without altering the underlying genome. These modifications can have a wide range of effects on gene expression, including genes activation or repression, thus modulating the immune response.

Until recently, it was commonly accepted that training and memory abilities were the hallmarks of adaptive immunity. However, several discoveries have challenged this paradigm.

Indeed, it has been shown that exposure to a pathogen could modulate the long-term immune response via the functional reprogramming of innate immune cells. This hypothesis was based on results showing that BCG vaccination surprisingly led to an increased long-term response to β-1,3-D-glucan in the wall of Candida albicans [99]. This phenomenon was mediated by specific and persistent changes in histone acetylation and methylation [100–103]. These findings allowed the identification of acquired and persistent immune response alteration, called innate immune memory, consisting either in an increased response to restimulation, called trained immunity, or a reduced immune response, called immune tolerance [104]. Finally, it should be noted that this phenomenon may directly concern the cells of innate immunity and their precursors, which can potentially transmit these alterations in immune memory [105].

Numerous inducers of immune training have been described based on experimental data. These different factors are thus likely to modify the phenotype of the response to aggression in case of prior exposure in ACI. Thus, we can mention Candida *albicans* and BCG as previously described, but also Mycobacterium *tuberculosis* [106], viral agents like HIV and HBV [107, 108], Plasmodium *falciparum* [109] or the diphtheria/tetanus/poliomyelitis/pertussis combined vaccine [110]. It is also interesting to note the potential influence of non-infectious factors on immune training, like diet [111], physical exercise [112], or circadian rhythm [113].

The pathways by which these different factors influence the long-term immune response have been widely studied. The activation of several metabolic pathways, including glycolysis, oxidative phosphorylation and lipid metabolism, plays a significant role [114–116]. These mechanisms generate numerous mediators which play a role in chromatin modifications involved in innate immune memory [116, 117].

From a clinical point of view, the innate immune training seems to be associated with a protective effect against specific pathogens. This was particularly illustrated during the COVID-19 pandemic. Indeed, several authors reported the potential protective role of BCG vaccination against SARS-CoV-2 infection [118, 119]. However, these beneficial effects seem to be counterbalanced by the existence of deleterious effects linked to innate immune memory, in the fields of chronic inflammatory diseases [120], allergology [121] or organ transplantation rejections [122].

### Age related features of innate immunity

#### Pediatric specificities

Infants’ immune system is still in development, and presents specific characteristics including impaired PAMPs and DAMPs recognition.

Two intrinsically linked mechanisms will be considered when discussing innate immunity in the intensive care units (ICU). The first question is the reason why some children have severe infectious diseases, with the hypothesis that such life-threatening disorders could be due to a pre-existing immune deficiency. The second is the consequences of ACI on the innate immune system.

Exploring how innate immunity deficiencies can lead to life-threatening acute illnesses in children permitted the identification of many immune deficiencies [123]. In young children, given the absence of a fully mature adaptive immunity, the innate immune system plays a crucial role in fending off infections. Indeed, rare deficiencies in the complement system have been shown to lead to disseminated meningococcal infection and/or meningitis [124]. In turn, such severe infection leads to uncontrolled inflammation and organ injuries. Similarly, in children with severe or critical COVID-19 pneumonia, deficiencies in the critical mediators of early antiviral defenses type I IFN have been shown to underlie more than 10% of cases [125, 126]. Moreover, in some children, inborn errors of OAS–RNase L can, following a trigger by SARS-CoV-2, unleash the production of MAVS-mediated inflammatory cytokines by mononuclear phagocytes, thus leading to a Kawasaki-like disease, called multi-inflammatory syndrome – children [127, 128].

ACI-associated innate immune disruption in children is hardly quantifiable as intimately intricated with the cause of severe disease. Nevertheless, in non-infectious settings in patients with acute organ damage in the ICU, several consequences on the innate immune system have been observed like immune paralysis [129] and reduction in key immune molecules such as HLA-DR, therefore facilitating the development of nosocomial infections [130].

Newborn children are a specific group as their immunity is developing and changing rapidly [131]. They are also highly susceptible to infections, for reasons that are not well understood [132].

Overall, identifying such immune deficiencies could guide the clinical strategy in children with ACI. It not only represents a challenge because of the consequences of inflammation or organ injuries, but more importantly because these deficiencies can be the potential underlying cause of the severe disease.

#### Immunosenescence

**Fig. 3:**
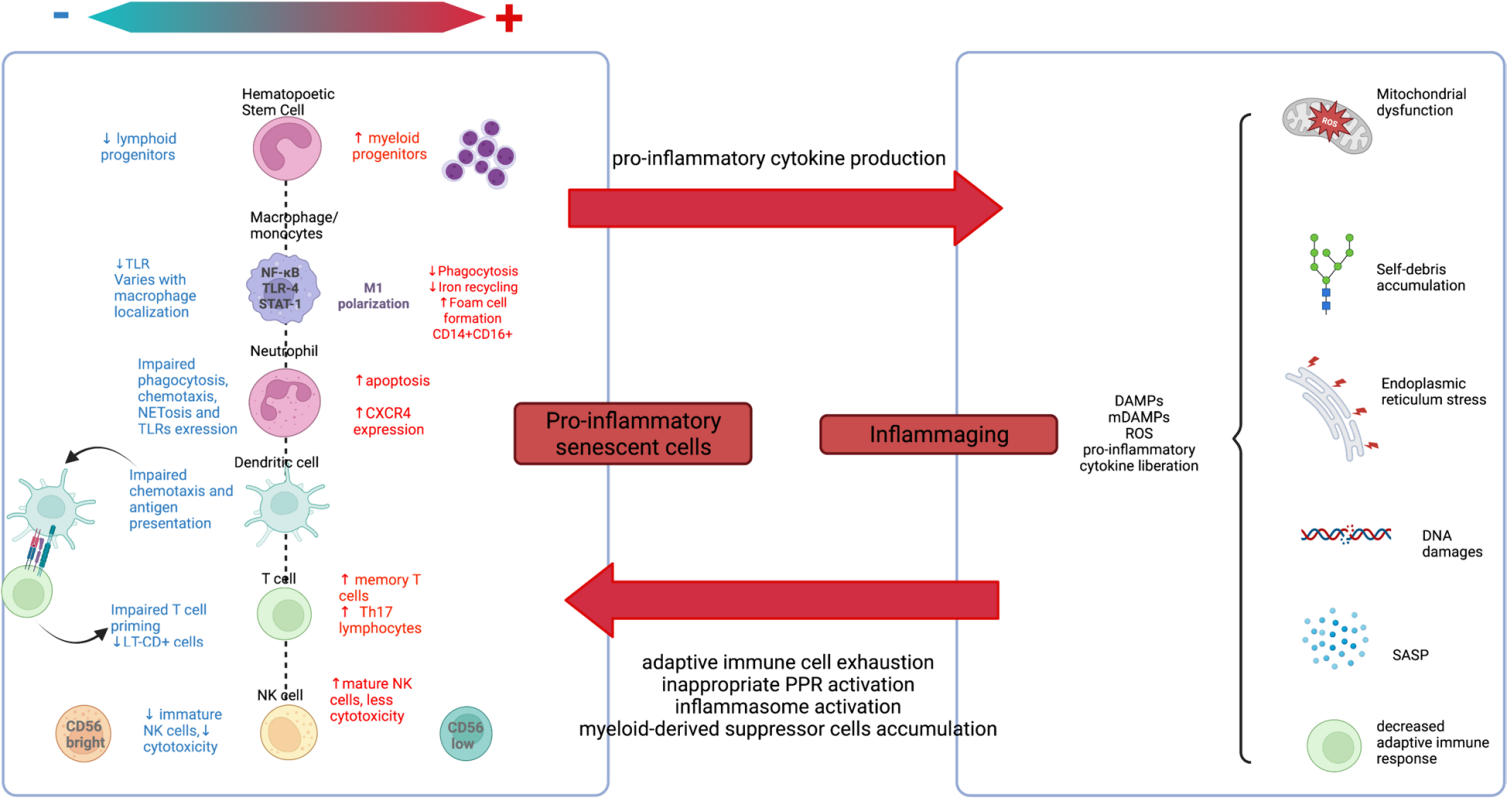
Innate immunity in aging patients. Balance between inflammatory and anti-inflammatory is highly modified in aging. The two main altered mechanisms are: a reduced capacity of response to injury by pro-inflammatory senescent cells (right) and a chronic systemic inflammation called inflammaging (left). *CD* cluster of differentiation, *DNA* desoxyribonucleic, *IL* interleukin, *LT* lymphocyte T, *NK* natural killer, *ROS* reactive oxygen species, *SASP* senescence-associated secretory phenotype, *Th* T helper, *TLR* toll like receptor, *TNF* tumor necrosis factor

Immunoscenescence refers to the progressive decline of immune functions with aging. The two pillars of this immune aging are a reduced capacity of response to pathogens and antigens -such as in vaccinations-, and a chronic low-grade systemic inflammation without evident trigger, called ‘inflammaging’ [133] (Fig. 3). The older population being heterogeneous, the level of immunosenescence and its consequences are highly variable between individuals of the same age.

##### Impact of aging on innate immune cells

Virtually all innate immune cells are affected with aging. First, HSC progenitors have a decreased functionality with altered expression and inappropriate activation of PRRs, and the distribution between lymphoid and myeloid progenitors is imbalanced, in favor of myeloid progenitors [133]. Regarding mature functional cells, the absolute number of innate immune cells shows little variations with age, but profound functional changes occur. Neutrophil have an increased clearance rate and their phagocytosis and chemotaxis capacities are reduced (after LPS stimulation) [134–136]. Monocytes display altered phenotypes along with functional changes: impaired phagocytosis, and impaired TLR1 and 2 pathways [137–140]. Macrophages display different alteration profile with ageing, according to their tissue residency and ontogeny [141, 142]. Their phagocytic capacities -including efferocytosis- appear impaired, but cytokine production might be increased in some conditions/subsets (thereby contributing to inflammaging), and reduced in others [143, 144]. DCs have also altered phagocytic and antigen presentation, and upon TLR stimulation, pro-inflammatory cytokine secretion is reduced, leading to impaired T cell priming and defective CD4 + T cell polarization [145].

Innate cells from lymphoid origin are also altered with ageing, though data are relatively scarce. Among ILCs, group 2 ILC from aging mice have been reported to have lower response during influenza infection [146]. Regarding ITCs, blood MAIT decreased in the blood during adulthood, potentially associated with functional alteration [147]. Similarly, blood levels of γδ T cells harboring Vδ2 chain decline with age andtheir phenotype is altered [148], but the clinical consequences of these observed phenomenon are still unknown.

##### Inflammaging

Modification of innate cells activation generates a dysregulated level of inflammation which participates in inflammaging characterized by a chronic increase in serum levels of pro-inflammatory cytokines such as IL-6, TNF-α, IFN-a and IL1-β [149]. Although still incompletely understood, this phenomenon results from several mechanisms: (i) inappropriate activation of PRRs by self-debris from defective autophagy, mitophagy and ubiquitin/proteasome system [150, 151], (ii) dysbiosis [152], (iii) mitochondrial dysfunction [153–155], (iv) cellular senescence with their “senescence-associated secretory phenotype” (SASP) and tissular aging [153, 156], (v) endoplasmic reticulum stress [157], (vi) and DNA damage [153]. Consequences include oxidative stress, tissular lesions, alteration of metabolism and endocrine system and accumulation of MDSCs. Inflammaging is associated with worse outcomes in older patients, with increased mortality, comorbidities, sarcopenia and frailty [158]. Inflammaging is not only a consequence of immunosenescence, as it plays a role in maintaining and aggravating immune cell senescence through adaptive immune cell exhaustion [159].

Inflammaging and altered innate immune cell function result in increased susceptibility to bacterial and viral infections. As a consequence, the incidence and severity of sepsis increases in the older population [160, 161]. Furthermore, some sepsis survivors have persistent inflammation reminiscent of accelerated immune aging, feeding a vicious cycle [162, 163].

No specific studies on therapies targeting the innate compartment during sepsis have been performed in the older population. Several have been explored, including immunoglobulins (to neutralize endotoxins and improve monocyte/macrophage phagocytic ability), IFN-g and GM-CSF (to enhance neutrophils and monocytes/macrophages phagocytosis, and cytokine release). To date, none have shown any efficacity on mortality [164]. Boosting immune system could be even deleterious [165, 166], and other clinical trials exploring the approach of immune boosting are underway.

### Immune response: behind the phenotype

Heterogeneity of the immune response remains as an important limiting factor for the development of targeted treatments in the field of acute inflammation. Recent data underline the difficulties of understanding the phenotypic specificities of each patient. Numerous studies have shown that patients who appear to be similar at bedside, due in part to similar sources of infection or type of aggression, may in fact differ in terms of the inflammatory response revealed by next-generation approaches [167]. The ability to group patient according to shared common pathophysiological processes could help to refine prognosis performance, and ultimately identify patients that could benefit from targeted therapies. Such categorization beyond clinical phenotypes is called ‘endotypes’ [168, 169]. The benefits of such an approach in patients characterization were notably highlighted by Antcliffe, et al., who showed that a transcriptomic analysis could distinguish subgroups of patients according to their degree of response to corticosteroid therapy during sepsis [170]. One important challenge in the field of critical illness remains to clearly define the scope of application of these new approaches, in order to better personalize the management of patients. Specifically, given importance of dysregulation of immune response in ACI, researchers should focus on defining immune endotypes from translational immunology discoveries in ACI. However, one of the major drawbacks for these potential therapeutic avenues is the lack of consideration of spatial dynamics and spatial specificities of innate immune response during ACI. Spatial considerations of immune response refer to the concept of compartmentalization of immune response: immune response to aggression, at a considered time-point, might differ in intensity and modality across anatomical sites of the body. This especially relevant in many situations in critical care where initial insult is frequently located at a given site, with subsequent systemic diffusion of inflammatory response. Thus, we and others demonstrated that during pneumonia-driven ARDS, inflammatory response -explored through cytokines concentrations- was largely compartmentalized to the lung [171, 172]. Moreover, beyond consideration of soluble mediators, cellular actors of innate immune response are particularly subject to phenotypic and functional alteration according to the organ considered (example: blood monocyte *versus* various multiple macrophages subsets). Consequently, sampling blood to monitor immune response might not be adequate to infer immune status of other distant anatomical sites [173].

## Conclusion

The ACI-associated systemic inflammatory response reflects the complex and highly dynamic host response mediated by the innate immune system. The overactivation of the immune response, partly responsible for critical illness, notably results from the synergy between the various mechanisms involved in the inflammatory response leading to its disproportionate amplification [174, 175].

In recent decades, milestones have been reached in understanding this response and its consequences in acute and chronic organ dysfunction. Some of these knowledge have already been translated into clinical trials targeting specific host pathways [176, 177]. However, the diversity of the underlying diseases and the highly variable host response makes the bridge from bench to bedside highly hazardous. Thus, many efforts have still to be made before considering future routine implementation of targeted host modulation strategies in critical care medicine. It is assumable that building new tools allowing precise real-time endotyping of the individual innate response during ACI are a preliminary necessary step before unleashing personalized immunomodulation of the innate response as part of resuscitation strategies.

## Data Availability

Not applicable.

## Abbreviations

ACI: Acute critical illness
AMPK: AMP-activated protein kinase
ARDS: Acute respiratory distress syndrome
BCR: B cell receptors
C1: Complement 1
CD: Cluster of differentiation
COX: Cyclooxygenase
CLP: Common lymphoid progenitor
CRP: C-reactive procedure
DAMPs: Danger-associated molecular patterns
DCs: Dendritic cells
DHA: Docosahexaenoic acid
DNA: Desoxyribonucleic
EPA: Eicosapentaenoic acid
HLA: Human leucocyte antigen
HSCs: Hematopoietic stem cells
ICU: Intensive care unit
ILC: Innate lymphoid cells
IFN: Interferon
IL: Interleukin
ITCs: Innate T cells
LK: Leukotriene
LTCI: Long-term cognitive impairment
LPS: Lipopolysaccharide
LT: Lymphocyte T
MAIT: Mucosal-associated invariant T
MDSC: Myeloid-derived suppressive cells
MVs: Microvesicles
NETs: Neutrophil extracellular traps
NK: Natural killer
NLRs: NOD-like receptors
NOD: Nucleotide-binding oligomerization domain
PAF: Platelet-activating factor
PAMPs: Pathogen-associated molecular patterns
PLA2: Phospholipase 2
PG: Prostaglandin
PRMs: Recognition molecules
PRRs: Pattern recognition receptors
PTX: Pentraxin
ROS: Reactive oxygen species
SASP: Senescence-associated secretory phenotype
SPM: Specialized pro-resolving mediators
TCR: T cell receptors
Th: T helper
TF: Tissue factor
TLC: Total lung capacity
TLR: Toll like receptor
TNF: Tumor necrosis factor
TXA: Thromboxanes (TXA)
